# 

**Published:** 1888-02-25

**Authors:** 


CONTENTS.

**The Principles of Progress** - - - - - - 359

**Words of Consolation.** Chapter LXVII.—The Sufferings of Christ - - - - - - - - 360

**The Doctor's Pulpit.**—The Science of Bedroom Fires - - 361

**Scotch Physicians and Surgeons.**—By C. G. Furley.—Sir Robert Christison - - - - - - - 362

**Aids to Nursing.** By M. M. Basil, M.A. M.B., Author of "The Commoner Accidents to Life and Limb" - - 363

**Round about the Asylums.**—By a Medical Superintendent. American Reports: State Lunatic Hospital at Harrisburg, Penn.; Lunatic Hospital, Northampton, Mass.; Lunatic Hospital, Taunton, Mass.; The State Asylum, Buffalo - - 365

**Notes and News** - - - - - - - - 366

**Everybody's Page** - - - - - - - - 368

**Annotations.**—Wicked or Weak?—Cock Fighting.—Early Marriages - - - - - - - - 369

**Rachel Challice's Vow.** By Lina Nevill, Author of "A Future on Trust," etc. - - - - - - 370

**Turning over New Leaves** - - - - - - 371

**The Nursing Mirror.**—Difficult Cases - - - - 373

**The Editor's Letter-box.**—The Dismissed Paris Nurses.—Free Nursing for the Poor - - - - - - 373

**Letter from Across the Sea** - - - - - - 374

**Amusements with Profit for all Ages.**—For Men and Women.—Children's Corner.—Puzzles, etc. - - - 374

